# HTLV-1 contains a high CG dinucleotide content and is susceptible to the host antiviral protein ZAP

**DOI:** 10.1186/s12977-019-0500-3

**Published:** 2019-12-16

**Authors:** Paola Miyazato, Misaki Matsuo, Benjy J. Y. Tan, Michiyo Tokunaga, Hiroo Katsuya, Saiful Islam, Jumpei Ito, Yasuhiro Murakawa, Yorifumi Satou

**Affiliations:** 1 Division of Genomics and Transcriptomics, Joint Research Center for Human Retrovirus Infection, Kumamoto University, Kumamoto, 860-0811 Japan; 2 International Research Center for Medical Sciences (IRCMS), Kumamoto University, Kumamoto, Japan; 3 Division of Hematology, Respiratory Medicine and Oncology, Department of Internal Medicine, Faculty of Medicine, Saga University, Saga, Japan; 4 Division of Systems Virology, Department of Infectious Disease Control, International Research Center for Infectious Diseases, Institute of Medical Science, The University of Tokyo, Tokyo, Japan; 5 RIKEN-IFOM Joint Laboratory for Cancer Genomics, RIKEN Center for Integrative Medical Sciences, Kanagawa, Japan

**Keywords:** Retrovirus, HTLV-1, HIV-1, Retroviral latency, Post-transcriptional regulation, ZAP

## Abstract

**Background:**

Human T cell leukaemia virus type 1 (HTLV-1) is a retrovirus associated with human diseases such as adult T-cell leukaemia/lymphoma and HTLV-1 associated myelopathy/tropical spastic paraparesis. In contrast to another human retrovirus, human immunodeficiency virus type 1 (HIV-1), HTLV-1 persists in the host not via vigorous virus production but mainly via proliferation and/or long-term survival in the form of silent proviruses in infected host cells. As a result, HTLV-1-infected cells rarely produce virus particles in vivo even without anti-retroviral treatment. That should be an advantage for the virus to escape from the host immune surveillance by minimizing the expression of viral antigens in host cells. However, why HIV-1 and HTLV-1 behave so differently during natural infection is not fully understood.

**Results:**

We performed cap analysis of gene expression (CAGE) using total RNAs and nascent, chromatin-associated, RNAs in the nucleus and found that HTLV-1 RNAs were processed post-transcriptionally in infected cells. RNA processing was evident for the sense viral transcripts but not the anti-sense ones. We also found a higher proportion of CG di-nucleotides in proviral sequences of HTLV-1-infected cells, when compared to the HIV-1 genomic sequence. It has been reported recently that CG dinucleotide content of viral sequence is associated with susceptibility to the antiviral ZC3HAV1 (ZAP), suggesting the involvement of this protein in the regulation of HTLV-1 transcripts. To analyse the effect of ZAP on HTLV-1 transcripts, we over-expressed it in HTLV-1-infected cells. We found there was a dose-dependent reduction in virus production with ZAP expression. We further knocked down endogenous ZAP with two independent targeting siRNAs and observed a significant increase in virus production in the culture supernatant. Other delta-type retroviruses such as simian T-cell leukaemia virus and bovine leukaemia virus, also contain high CG-dinucleotide contents in their viral genomes, suggesting that ZAP-mediated suppression of viral transcripts might be a common feature of delta-type retroviruses, which cause minimal viremia in their natural hosts.

**Conclusions:**

The post-transcriptional regulatory mechanism involving ZAP might allow HTLV-1 to maintain a delicate balance required for prolonged survival in infected individuals.

## Background

The human T cell leukaemia virus type 1 (HTLV-1) was the first retrovirus that was associated with a human disease [1–3]. Specifically, it causes adult T-cell leukaemia/lymphoma (ATL) and several inflammatory diseases such as HTLV-1-associated myelopathy/tropical spastic paraparesis [4–7]. Further, this virus infects approximately 20 million people worldwide, and mainly those living in endemic areas including Southwestern Japan, the Caribbean, and sub-Saharan Africa [8]. As a retrovirus, HTLV-1 integrates into the genome of infected cells in the form of a provirus. The plus and minus strands of this provirus encode several viral proteins, such as Tax and HBZ [9]. Another retrovirus, human immunodeficiency virus type I (HIV-1), show vigorous viral replication without anti-retroviral drugs, but HTLV-1 persists in infected individuals without virus in the plasma even in the absence of anti-retroviral drugs. It has also been reported that some HIV-1-infected clones expand clonally like HTLV-1-infected cells [10, 11]. Most of them carry defective proviruses [12], whereas HTLV-1-infected clones carrying full-length provirus seems to expand without producing viral particles, suggesting latency-prone phenotype of HTLV-1-infected cells. Most of HTLV-1-infected cells are transcriptionally silenced in vivo, but they quickly increase a few hours after culture ex vivo [13, 14]. In contrast, the minus-strand transcript HBZ is present in the majority of infected cells, at low levels [15, 16]. This pattern of viral gene expression is regulated by a complex mechanism involving cellular, viral, and metabolic factors [17, 18]. For example, at the post-transcriptional level, the plus-strand-encoded Rex viral protein plays an important role in orchestrating the nuclear export of viral mRNAs [19]. In addition, it has been reported that HBZ mRNA is retained in the nucleus [20]. These indicates there would be unidentified regulatory mechanisms for proviral transcriptional regulation.

Microbial infections are detected by the host through multiple mechanisms. Viruses can be recognized by pattern recognition receptors such as RIG-I, which trigger an intracellular signalling cascade activating the expression of inflammatory mediators to eliminate infected cells and pathogens [21]. In addition to these transmembrane and cytoplasmic receptors, viral infections can also be inhibited by the action of cellular restriction factors at different stages in their life cycle [22]. Among these restriction factors, the ZC3HAV1 (ZAP) protein has been reported to exert antiviral activity against a broad range of viral families including alphaviruses, filoviruses, Hepatitis B virus, influenza A virus, and retroviruses such HIV-1 [23]. It still remains largely unknown how these antiviral mechanisms control persistence of HTLV-1 infection in the host [24]. In this study, we sought to determine whether HTLV-1 viral transcripts could be detected by ZAP, targeting them for degradation or processing.

## Results

### The cap analysis of gene expression (CAGE) profile of an HTLV-1-infected cell line suggests the processing or degradation of viral RNAs

**Fig. 1:**
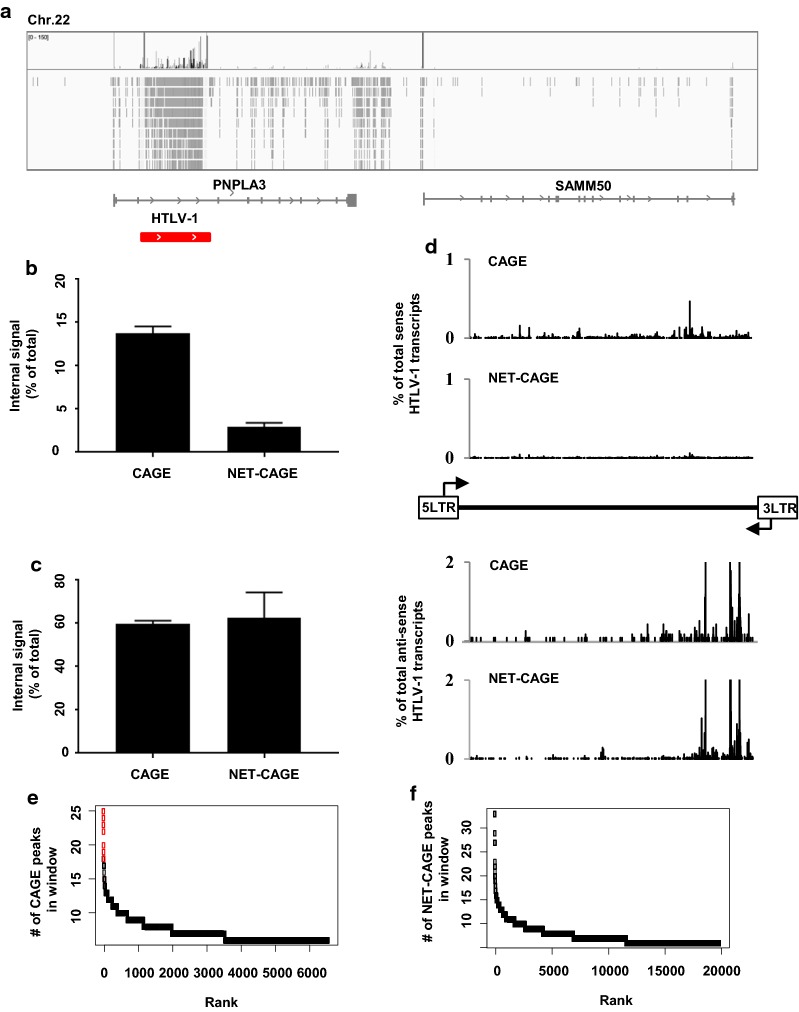
Evidence for RNA processing in an HTLV-1-infected cell line. **a** One representative cap-associated gene expression (CAGE) profile for TBX-4B cells is visualized by integrate genomic viewer (IGV). Each gray line represents each sequencing read we detected in the analysis. The region around the integrated provirus is shown as red bar. **b** Quantification of the CAGE and nascent-elongating-transcript CAGE (NET-CAGE) reads in the sense orientation within the proviral region, between the LTRs. **c** The same is shown for reads in the anti-sense orientation. **d** CAGE and NET-CAGE profiles in the proviral region between the LTRs (top: sense signal; bottom: anti-sense signal). Only the first nucleotide of the 5′ side of the transcripts is shown (transcription start site; TSS). The signal was calculated as a percentage of total reads that aligned to the provirus. **e** Signal in a sliding window of 10 kb is shown for CAGE. **f** NET-CAGE datasets of TBX-4B cells. Red circles correspond to virus-aligned reads, whereas black circles correspond to human genome-aligned reads

The expression of HTLV-1 transcripts occurs in bursts or intermittently in infected cells [25, 26], indicating that the regulation of HTLV-1 transcripts at the transcriptional or post-transcriptional levels is more complex than our current understanding. To gain insight into the pattern of proviral transcript regulation in more detail, we conducted CAGE [27], which detects 5′ capped RNAs and is therefore useful to identify 5′ end of RNAs and transcription start sites (TSSs), as well as to quantify coding and non-coding RNAs with a 5′-cap structure. We analysed the TBX-4B cell line, isolated from the peripheral blood of an HTLV-1-infected individual, which contains one copy of the integrated provirus in chromosome 22; moreover, this provirus is highly transcribed (Fig. 1a) [28]. The level of transcription in the sense direction in these cells was much higher than that in the antisense direction (Additional file 1: Fig. S1). In general, CAGE signals tended to accumulate near the TSS, as observed for the host genes *PNPLA3* and *SAMM50* (Fig. 1a). Unexpectedly, the CAGE signal was not only detectable in the LTRs, which serve as promoters, but was broadly spread all along the provirus (Fig. 1a). These data suggested two possibilities. First, there might be cryptic proviral transcription from the region within the 5′ and 3′ LTRs. Second, HTLV-1 RNAs might be post-transcriptionally degraded, resulting in a broad CAGE signal when cleaved RNAs are re-capped. Previous studies reported that some CAGE tags align to not only TSSs or enhancer regions but also other genomic regions such as exonic regions, indicating that transcribed RNAs are processed and recapped and thereby detectable by CAGE [29, 30]. To distinguish these two possibilities, we performed a modified version of CAGE, called native elongating transcript-CAGE (NET-CAGE), where nascent RNAs that are not yet affected by post-transcriptional processing are purified from chromatin and used as an input for CAGE [31]. We compared the ratio of signals in the internal region of the provirus between CAGE and NET-CAGE, and found that with NET-CAGE this was much lower than that with CAGE in the sense direction (Fig. 1b). This tendency was not observed in the antisense direction (Fig. 1c). We calculated the proportion of CAGE signals in the internal region of the provirus within the total number aligning to the whole provirus and plotted the results for the plus and minus strands separately (Fig. 1d). We observed a larger number of peaks in CAGE than in NET-CAGE for plus-strand-aligning reads (Fig. 1d, top). This difference was not observed for the minus strand-aligning reads (Fig. 1d, bottom). It has been reported that broad CAGE signals occur around the TSSs of human genes with high levels of transcription [29]. We searched the whole human genome for a high-density CAGE signal in TBX-4B cells. We performed a sliding window analysis to investigate the number of CAGE peaks present in respective 10-kb windows, which is approximately the size of the provirus. We discovered that the integrated HTLV-1 provirus was the most significantly enriched region for multiple CAGE signals within the human genome, as shown by the red circles in Fig. 1e. This tendency was not observed with NET-CAGE (Fig. 1f), suggesting that 5′-capped RNAs originating from the internal regions of the provirus are rare in the nucleus but present in the cytoplasm. These findings indicate that the broad CAGE signal observed in the internal proviral region (Fig. 1a) are generated at the post-transcriptional level.

### The proviral sequences of delta retroviruses contain higher proportions of CG di-nucleotides

**Fig. 2:**
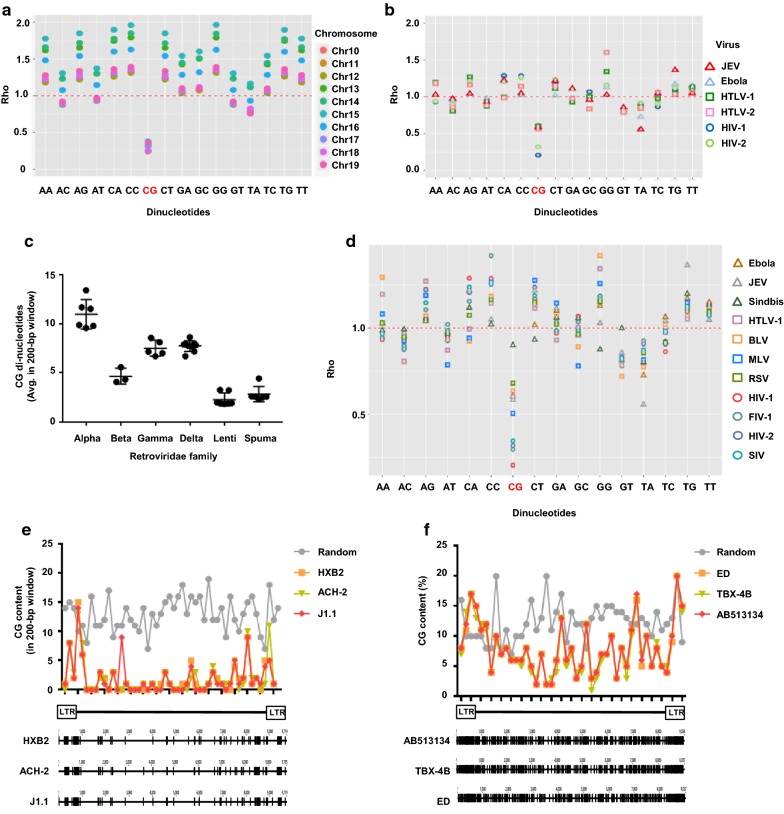
Delta retroviruses have a higher a CG content. **a** *ρ* statistic of each dinucleotide within the human genome, shown independently for some chromosomes. **b** The same analysis is shown for HIV-1, HIV-2 (lentiviruses), HTLV-1, HTLV-2 (delta retroviruses), JEV, and Ebola (previously reported to be ZAP targets). **c** CG dinucleotide content is shown for several retroviruses grouped by families. **d** *ρ* statistic of each dinucleotide within retroviruses and viruses reported to be ZAP targets. Proportion of CG-dinucleotides in a 200-nucleotide window in viral (HIV-1: **e**, HTLV-1: **f**) and a random sequence. Below each graph, individual proviral sequences are shown, wherein black lines represent a CG di-nucleotide

Several cellular proteins target viral RNA, relying on different mechanisms to detect them and mount an anti-viral immune response. ZAP is a cellular restriction factor that shows antiviral activity against a wide range of RNA viruses [23, 32–35]. In addition to a complex secondary structure [36, 37], a high content of CG di-nucleotides seems to be a key factor for the recognition of targets by ZAP [38]. The evolution of RNA viruses of vertebrates, such as HIV-1, has been reported to lead to viral genomes with a low proportion of CGs to escape from the anti-viral host mechanism [38, 39]. We analysed the proportions of various dinucleotides in the human genome and found that the CG dinucleotide was under-represented compared to other dinucleotides, consistent with previous reports [39, 40] (Fig. 2a). We next performed the same analysis for several retroviruses and found that CG suppression in HIV-1 and HIV-2 was similar to that of the human genome (Fig. 2b), but was not as apparent in HTLV-1 and HTLV-2. The same tendency was observed for other delta retroviruses and previously-reported ZAP-target viruses (Fig. 2c, d). We then analysed the CG di-nucleotide content and distribution along the provirus based on three HIV-1 and three HTLV-1 sequences (Fig. 2e, f). A sequence of approximately the same length as HIV-1 and HTLV-1, 10 kb and 9 kb, respectively, with a random distribution of CG di-nucleotides, was used for comparison. On one hand, there was a clear depletion of CG dinucleotides in HIV-1 genomes (Fig. 2e). On the other hand, the depletion of the CG di-nucleotides was not as evident in HTLV-1 sequences compared to that in the random control (Fig. 2f). These data demonstrated that HTLV-1 maintains a high CG dinucleotide content despite long-term co-existence with humans [41].

### HTLV-1 viral transcripts contain a higher proportion of CG di-nucleotides and could be targeted by ZAP

**Fig. 3:**
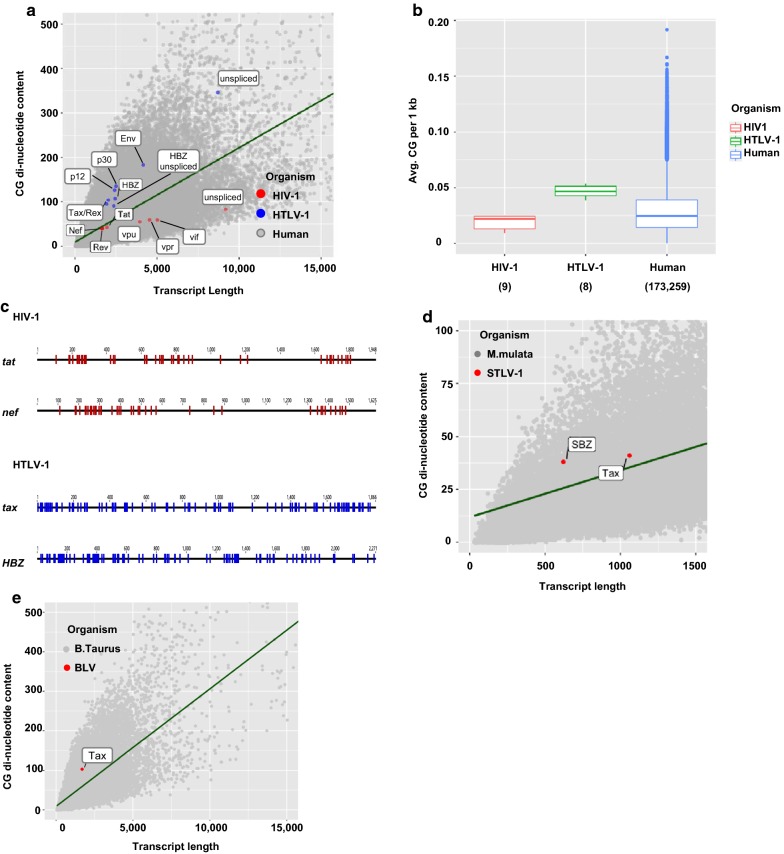
HTLV-1 transcripts show a higher content of CG di-nucleotides. **a** The CG dinucleotide content of HIV-1 (red dots), HTLV-1 (blue dots), and human (grey dots) transcripts is shown in correlation with the transcripts’ lengths. HIV-1 mRNAs used in this analysis are shown in Additional file 1: Table S4 [56]. **b** The average number of CG di-nucleotides per kb of transcript is shown for the same organisms. Numbers in parentheses indicate the total number of transcripts considered for the calculation. **c** Individual CG dinucleotides are shown for viral transcripts. Blue: HTLV-1, red: HIV-1. **d** Proportion of CG-dinucleotides in relation to transcript length for STLV-1 (NCBI GenBank: JX987040.1) and monkey (*Macaca mulatta*) genomes. To visualize the difference between average value and STLV-1 Tax, expanded version of the graph is shown. **e** Proportion of CG-dinucleotides in relation to transcript length for BLV and bovine (*Bos taurus*) genomes

The HTLV-1 genome encodes several viral RNAs both in sense and antisense orientations [42]. We next analysed the CG dinucleotide content of HIV-1 and HTLV-1 transcripts together with the those of the host cell. The CG dinucleotide content per transcript length for all HTLV-1 transcripts was higher than the average value for human transcripts. In contrast, that of all HIV-1 transcripts was lower than the average value for human transcripts (Fig. 3a, b). CG di-nucleotides were found to be distributed broadly in the HTLV-1 transcripts *tax* and *HBZ* compared to distribution in HIV-1 transcripts such as *tat* and *nef* (Fig. 3c). Transcripts of simian T-cell leukaemia virus type 1 (STLV-1) and bovine leukaemia virus (BLV) also showed a higher CG content compared to those of their hosts (Fig. 3d, e, respectively).

### ZAP exerts an inhibitory effect on the production of HTLV-1

**Fig. 4:**
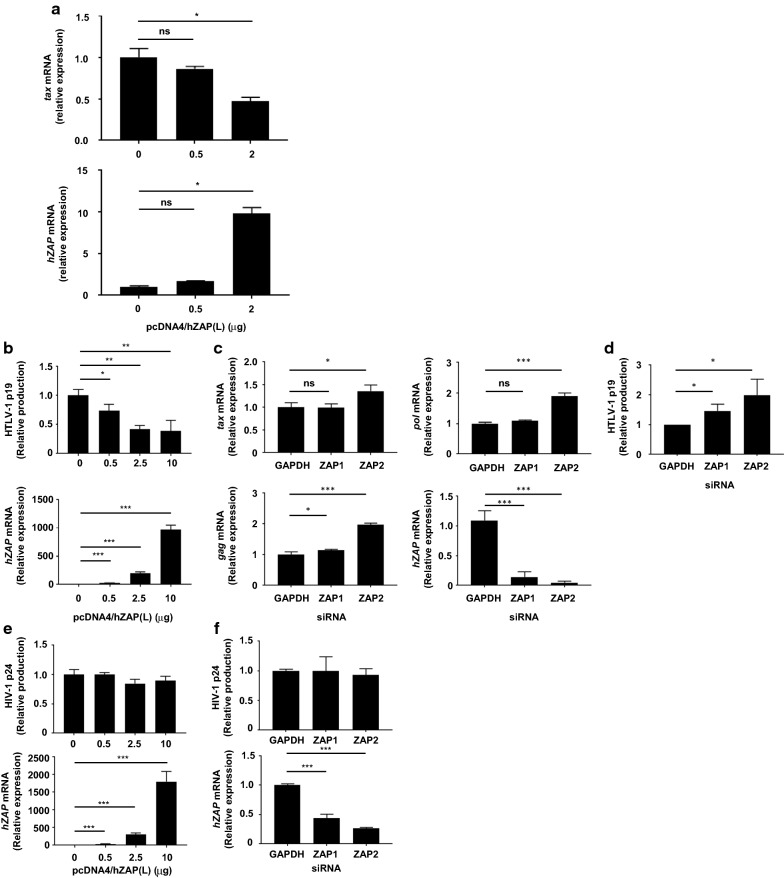
The involvement of ZAP in the regulation of HTLV-1 production. **a** HeLa cells were transfected with a Tax expression vector in the presence of increasing amounts of a ZAP expression vector. Changes in the level of *tax* transcripts (top) were measured by RT-qPCR, in addition to those of *hZAP* (bottom). *p < 0.0001. **b** JEX22 cells were transfected with increasing amounts of a human ZAP expression vector and cultured for 24 h. Stimulation with PMA/ionomycin was performed for 4 h before collecting the supernatant and cells for analysis. **c**, **d** JEX22 cells were transfected with the indicated siRNAs and cultured for 24 h. They were then stimulated, as indicated previously in this figure, before analysis. HTLV-1 RNAs (**c**) or p19 protein (**d**) was was measured (top panel) upon knock-down of endogenous ZAP (bottom panel). **e** J1.1 cells were transfected with increasing amounts of a human ZAP expression vector and cultured for 24 h. Stimulation with TNFα was performed for 4 h before collecting the supernatant and the cells for analysis. **f** J1.1 cells were transfected with the indicated siRNAs and cultured for 24 h. They were then stimulated as indicated before analysis. No significant changes were observed in p24 protein levels in the supernatant (top panel) upon knock-down of endogenous ZAP (bottom panel). *p < 0.05; **p ≤ 0.001; ***p < 0.0001

Next, we made a hypothesis that HTLV-1 transcripts are targeted by ZAP because of their high GC content. To test this, we analysed the effect of ZAP expression on the HTLV-1 transcript *tax*. We transfected HeLa cells with Tax and ZAP expression vectors and found that ZAP expression decreased Tax RNA level in a dose-dependent manner (Fig. 4a). The effect was modest but statistically significant. These data demonstrated that HTLV-1 viral transcripts contain a high proportion of CG di-nucleotides, and could be targeted by ZAP. To evaluate the role of ZAP in HTLV-1 production, we over-expressed ZAP using a ZAP-expression vector to transfect JEX22 cells, a cell line latently infected with HTLV-1. This cell line expresses viral transcripts upon stimulation with PMA/ionomycin. The over-expression of ZAP decreased the production of HTLV-1 p19 protein in the culture supernatant in a dose-dependent manner (Fig. 4b). To investigate the role of ZAP under physiological conditions, we next knocked down endogenous expression using siRNAs in JEX22 cells. ZAP knock-down using two different siRNAs significantly reduced the level of some sensei viral RNAs, such as tax, gag, and pol region (Fig. 4c), as well as virus production in the culture supernatant (Fig. 4d). The better ZAP suppression by ZAP2 siRNA resulted in the enhanced production of p19 compared to that with ZAP1 siRNA. These results indicate that ZAP expression negatively regulates virus production in HTLV-1-infected cells. We also performed ZAP over-expression and knock-down experiments using the HIV-1-infected cell line J1.1. There were no significant changes in p24 expression with either ZAP over-expression or knock-down (Fig. 4e, f, respectively). These data demonstrate that HTLV-1 is susceptible to the host cellular defence mechanism mediated by ZAP, possibly due to the high CG content of the viral genome sequence.

## Discussion

During HTLV-1 natural infection, there is no detectable viremia in the peripheral blood of infected individuals, even in the absence of anti-retroviral drugs. This is in sharp contrast to that with another human retrovirus, HIV-1, in which viral latency is rare in the absence of anti-retroviral drugs. It seems that HIV-1 and HTLV-1 evolved quite differently in terms of strategies to achieve persistent infection in the host. Both HIV-1 and HTLV-1 target CD4^+^ T cells. However, HIV-1 induces apoptosis in infected cells through vigorous virus production, which suppresses the anti-viral immune response and enables the virus to achieve persistent infection in the host. In contrast, HTLV-1 rarely produces viral particles, but rather promotes the proliferation and survival of infected cells to maintain a viral reservoir in the host. These different viral strategies to achieve persistent infection are associated with viral pathogenesis. HIV-1 induces acquired immunodeficiency syndrome by depleting infected CD4^+^ T cells, whereas HTLV-1 leads to the development of leukaemia in HTLV-1-infected cells in some infected individuals after a long latency, as a consequence of the enhanced proliferation and/or survival of infected CD4^+^ T cells. Several reasons have been proposed as to explain the enhanced latency of HTLV-1, such as different activity of the viral LTR promoters and the presence of antisense transcripts in HTLV-1 [42–44].

In this study, we identified another mechanism that could explain why HTLV-1 is prone to latency in vivo. The anti-viral systems of the host cell are negative regulators of viruses. However, HTLV-1 seems to take advantage of its high susceptibility to the host antiviral system, specifically ZAP-mediated viral RNA processing, to minimize viral antigen expression and thereby maintain latent infection. ZAP-mediated RNA processing is also a type of restriction factor for these viruses. Based on our findings including an abnormal CAGE pattern (Fig. 1) and high CG-dinucleotide content in the HTLV-1 genome (Fig. 2), we suggest that ZAP might regulate HTLV-1 transcripts at the post-transcriptional level. CAGE is a type of RNA-seq that facilitates the identification of the 5′ end of an RNA molecule by capturing its 5′ cap structure. In principle, we can detect both coding and non-coding 5′-capped RNA. Our CAGE and NET-CAGE results indicated that the broad CAGE signals in the internal proviral region result from RNAs present in the cytoplasm but not in the nucleus. This suggests the possibility that HTLV-1 RNA is processed at least partially mediated by ZAP, can be re-capped in the cytoplasm and thereby detected only by CAGE.

It has been reported that CAGE signals can cross exon–exon junctions, and therefore, these must have arisen from at least partially processed mRNAs [29]. Our data also indicate that HTLV-1 transcripts are processed at least partially by ZAP and can be recapped by an unknown mechanism and thereby detected by CAGE.

It has been reported recently that HTLV-1 transcription in the sense orientation is only intermittently active in an ATL cell line and primary infected T-cell clones [25, 26]. Further, there is a strong burst of proviral sense transcription but the expression is transient and is terminated spontaneously. Glucose metabolism and oxygen availability play a role in the reactivation of proviral expression from latency [45]; however, how such HTLV-1 transcriptional bursts terminate remains elusive. Since ZAP expression is induced by viral infection via an IRF-3-dependent pathway [46], the HTLV-1 burst might trigger ZAP expression. Then induced ZAP might target HTLV-1 transcripts as a negative feedback mechanism. It has been reported that promoter-associated small RNAs generated from the TSS of the *c*-*Myc* gene suppress *c*-*Myc* messenger RNA abundance [29]. Thus, processed viral RNA might not be just a consequence of RNA degradation but also could have a regulatory function to control HTLV-1 production at the post-transcriptional and translational level.

The abundance of antisense transcript CAGE signals was not significantly different between conventional CAGE and NET-CAGE (Fig. 1c), although both sense and antisense transcripts were found to contain a high CG-dinucleotide content. The CG-dinucleotide content not only determines ZAP susceptibility, but other factors such as the secondary structure of RNA are also involved [36, 37]. The abundance of transcripts might also play a role in susceptibility to ZAP; therefore, the low level of antisense transcripts in TBX-4B cells makes them less sensitive to ZAP. It has been reported that antisense proviral transcription is constitutively active, whereas sense transcription is frequently suppressed or expressed only intermittently. Previous studies also reported that transcription is regulated by genetic and epigenetic mechanisms [47–50]; however, the findings of the current study indicate that there is another regulatory mechanism controlling proviral transcription at the post-transcriptional level.

## Conclusion

HTLV-1 has evolved to maintain latency via multiple mechanisms. We show in this study that post-transcriptional RNA processing via antiviral ZAP is an additional strategy through which HTLV-1 achieves persistent infection in the host.

## Methods

### Cell lines

TBX-4B [28], an HTLV-1-infected clone derived from PBMCs of a HAM/TSP patient, was kindly provided by Dr. Charles Bangham (Imperial College London). These cells were cultured in RPMI supplemented with 20% FBS (SIGMA), 200 U/ml human recombinant IL-2 (Wako), 100 U/ml penicillin (Nacalai Tesque), and 100 μg/ml streptomycin (Nacalai Tesque). TBX-4B contains one copy of the integrated provirus in chromosome 22. JEX22 cells are latently infected with HTLV-1, and were kindly provided by Dr. Jun-ichi Fujisawa (Kansai Medical University). These cells contain two copies of integrated proviruses, in chromosomes 4 and 16. HeLa, a human adenocarcinoma cell line, was also used for transfection experiments. J1.1cells were obtained through the AIDS Research and Reference Reagent Program, Division of AIDS, NIAID, NIH from Dr. Thomas Folks [51]. These cells were handled in a bio-containment level 3 room. Except for TBX-4B cells, which were cultured as detailed, all other cells were cultured in RPMI supplemented with 10% FBS, 100 U/ml penicillin (Nacalai Tesque), and 100 μg/ml streptomycin (Nacalai Tesque).

### Proviral DNA sequence analysis

The proviral sequences of several retroviruses were obtained from PubMed. Accession numbers are provided in Additional file 1: Table S1. The number CG di-nucleotides was counted using Geneious (Biomatters Ltd.) software, which was also used to generate the schematic representation of their distribution within the proviral sequences. The number of CG di-nucleotides for each virus was plotted in graphs generated with GraphPad software. For comparison, a 9040 nucleotide-long random sequence was generated with the following website: http://www.faculty.ucr.edu/~mmaduro/random.html (https://www.bioinformatics.org/sms2/random_dna.html). The *rho* statistic (*ρ*) was computed for each dinucleotide pair using the R library *seqinr*. In brief, *ρ* measures how over- or under-represented a particular DNA nucleotide is and for a DNA dinucleotide, *ρ* is defined as:

$$\rho \left( {xy} \right) = \frac{{f\left( {xy} \right)}}{f\left( x \right) \times f\left( y \right)}$$

where f is the frequency of the nucleotide x/y/xy. *ρ* is expected to be equal to 1 when dinucleotide (xy) is formed by chance. If *ρ* is more than 1, the dinucleotide is much more common than expected, i.e. over-represented and vice versa.

### Bioinformatic analysis of human and viral transcripts

The correlation between CG di-nucleotide content and the length of the transcript was calculated and plotted in graphs for human, HIV-1, and HTLV-1 genes using the ggplot2 package in R. Datasets are provided in Additional file 1: Table S2.

### CAGE and NET-CAGE

Total and nascent RNAs were harvested from TBX-4B cells as previously described [31]. CAGE libraries were generated using CAGE library preparation kit (KK DNAFORM) following the manufacturer’s instructions. Briefly, first strand cDNA was synthesized from 5 μg of total RNA using random primers. The cap at the 5′end of the RNAs was biotinylated to facilitate the subsequent cap-trapping step. Remaining RNA fragments were digested with RNaseONE enzyme. Approximately 10 ng of each cDNA was used for linker ligation and library preparation. CAGE libraries were quantified by qPCR and size distribution was evaluated by TapeStation (Agilent Technologies) before sequencing in a NextSeq device (Illumina) as described previously [52]. NET-CAGE was performed as described previously [31]. We added the step to separate nuclear RNA and cytoplasmic RNA before we performed CAGE protocol.

### Data analysis for CAGE and NET-CAGE

Fastq files obtained from the sequencers were quality-checked and adaptor sequences were trimmed. Alignment to the human genome (hg19) and the HTLV-1 genome (Genbank, AB513134.1) was performed using the BWA-MEM algorithm with default parameters [53, 54]. TSSs were counted after obtaining the position of the first nucleotide of each read from the sequencing data.

### Knock-down and over-expression of ZAP

The knock-down of endogenous ZAP expression in JEX22 cells was carried out using two different siRNAs targeting the following sequences: GGUAAAACCUGGACGGACU (siZAP1) and GUGUAAGGGUUGUCCGCUU (siZAP2) [34]. siRNAs were transfected by electroporation into 2 × 10^6^ cells (NepaGene). After overnight culture, cells were stimulated for 4 h with PMA (50 ng/ml) and ionomycin (1 μM). The culture supernatant was collected to determine p19 presence by ELISA (RETROtek) in accordance with the manufacturer’s instructions and RNA was extracted using a RNeasy (Qiagen). To analyze the efficiency of knock-down of ZAP, 500–1000 ng of total RNA was used to synthesize cDNA, and ZAP expression was determined by RT-qPCR. HTLV-1 transcripts were also quantified by RT-qPCR. Results were calculated using the delta–delta CT method, normalising to 18SrRNA expression and comparing to ZAP expression in cells transfected with control siRNA (GAPDH). The sequences of the primers used are listed in Additional file 1: Table S3.

The same procedure was followed for ZAP knock-down in J1.1 cells, an HIV-1-infected cell line. For this, stimulation was performed with TNFα (10 ng/ml), and virus production was determined based on the presence of p24 antigen in the culture supernatant by ELISA (RETROtek) following the manufacturer’s instructions.

For ZAP over-expression experiments, JEX22 or J1.1 cells were electroporated with increasing amounts of a ZAP expression vector (Addgene number 45907). The following day, cells were stimulated with PMA/ionomycin or TNFα for 4 h. Culture supernatants were collected to measure the presence of viral antigens by ELISA, as described previously herein. RNA was extracted from the transfected cells to confirm the effective over-expression of ZAP.

### Over-expression of Tax and ZAP

HeLa cells (1.5 × 10^6^ cells/2 ml) were seeded in a 6-well plate, and the following day they were transfected with a Tax expression vector, pCG-Tax [55], in the presence of increasing amounts of a ZAP expression vector. Twenty-four hours later, RNA was extracted, and *Tax* transcripts were semi-quantified by RT-qPCR, in addition to *ZAP*, based the delta–delta CT method as reported previously [49].

## Supplementary information

**Additional file 1: Fig. S1.** CAGE result of TBX-4B cells.

**Additional file 2: Table S1.** Genome accession numbers. **Table S2.** Dataset accession numbers. **Table S3.** Primers and oligonucleotides used in this study. **Table S4.** HIV-1 sequences we analyzed in this study.

## Data Availability

The fastq files that were obtained in this study have been deposited in the DDBJ, DNA Data Bank of Japan (accession no. DRA009348). Data on the findings reported here are available from the corresponding author upon request.
