# Supplementary material for: HTLV-1 contains a high CG dinucleotide content and is susceptible to the host antiviral protein ZAP
Source: Retrovirology. 2019 Dec 16;16:38. doi: 10.1186/s12977-019-0500-3 (PMC6915898; doi:10.1186/s12977-019-0500-3)
Supplement: Supplementary file 1 — Additional file 1: Fig. S1. CAGE result of TBX-4B cells. [file 12977_2019_500_MOESM1_ESM.pptx]

## Slide 1
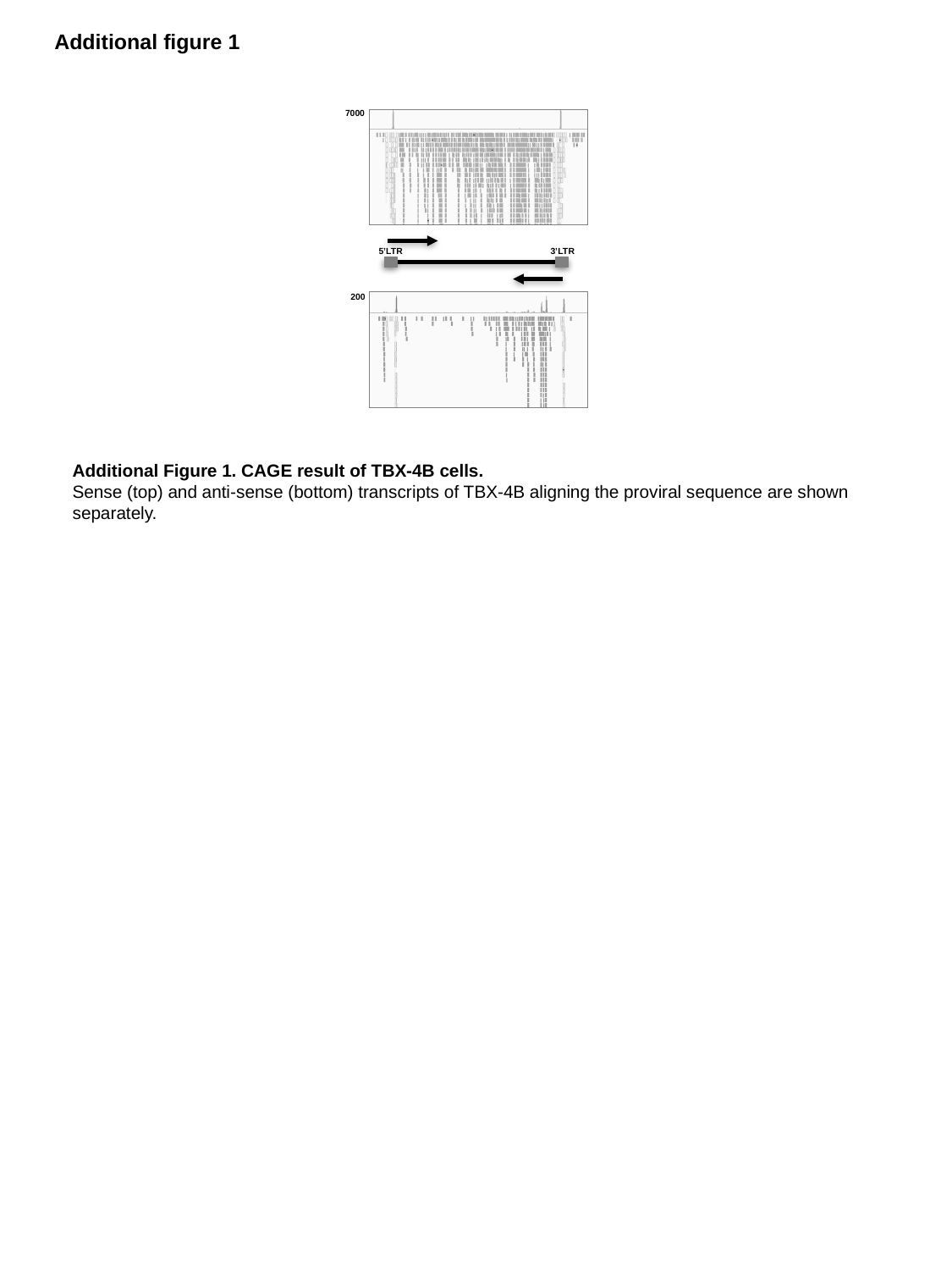

Additional figure 1
7000
5’LTR
3’LTR
200
Additional Figure 1. CAGE result of TBX-4B cells.
Sense (top) and anti-sense (bottom) transcripts of TBX-4B aligning the proviral sequence are shown separately.
