# Supplementary material for: HTLV-1 contains a high CG dinucleotide content and is susceptible to the host antiviral protein ZAP
Source: Retrovirology. 2019 Dec 16;16:38. doi: 10.1186/s12977-019-0500-3 (PMC6915898; doi:10.1186/s12977-019-0500-3)
Supplement: Supplementary file 2 — Additional file 2: Table S1. Genome accession numbers. Table S2. Dataset accession numbers. Table S3. Primers and oligonucleotides used in this study. Table S4. HIV-1 sequences we analyzed in this study. [file 12977_2019_500_MOESM2_ESM.docx]

**Additional table 1. Viral genome accession numbers**

| **Virus** | **Accession number** | **Family/Subfamily** |
| --- | --- | --- |
| Avian Leukemia Virus | NC_15116.1 | Retro. Alpha |
| Avian Leukosis Virus - RSA | NC_001408.1 | Retro. Alpha |
| Avian Sarcoma Virus CT10 | NC_038922.1 | Retro. Alpha |
| Fujinami Sarcoma Virus | NC_001403.1 | Retro. Alpha |
| Rous Sarcoma Virus | NC_001407.1 | Retro. Alpha |
| UR2 Sarcoma Virus | NC_001618.1 | Retro. Alpha |
| Jaagsiekte Sheep Retrovirus | NC_001494.1 | Retro. Beta |
| Mason-Pfizer Monkey Virus | NC_001550.1 | Retro. Beta |
| Mouse Mammary Tumor Virus | NC_001503.1 | Retro. Beta |
| Feline Leukemia Virus | NC_001940.1 | Retro. Gamma |
| Moloney Murine Leukemia Virus | NC_001501.1 | Retro. Gamma |
| Koala Retrovirus | NC_039228.1 | Retro. Gamma |
| Gibbon Ape Leukemia Virus | NC_001885.1 | Retro. Gamma |
| Reticuloendotheliosis Virus | NC_006934.1 | Retro. Gamma |
| Human T cell Leukemia Virus type 1 | AB513134.1 | Retro. Delta |
| Human T cell Leukemia Virus type 1 | NC_001436.1 | Retro. Delta |
| Human T cell Leukemia Virus type 2 | NC_001488.1 | Retro. Delta |
| Simian T cell Leukemia Virus type 1 | NC_000858.1 | Retro. Delta |
| STLV isolate baboon F88395 | JX987040.1 | Retro. Delta |
| Simian T cell Leukemia Virus type 2 | NC_001815.1 | Retro. Delta |
| Bovine Leukemia Virus | NC_001414.1 | Retro. Delta |
| Bovine Foamy Virus | NC_001831.1 | Retro. Spuma |
| Feline Foamy Virus | NC_039242.1 | Retro. Spuma |
| Japanese Macaque Simian Foamy Virus | NC_039026.1 | Retro. Spuma |
| Puma Feline Foamy Virus | NC_039022.1 | Retro. Spuma |
| Rhesus Macaque Simian Foamy Virus | NC_039238 | Retro. Spuma |
| Simian Foamy Virus | NC_031364.1 | Retro. Spuma |
| Human Immunodeficiency Virus type 1 | NC_001802.1 | Retro. Lenti |
| HIVHXB2CG | K03455.1 | Retro. Lenti |
| Human Immunodeficiency Virus type 2 | NC_001722.1 | Retro. Lenti |
| Simian Immunodeficiency Virus | NC_001549.1 | Retro. Lenti |
| Feline Immunodeficiency Virus | NC_001482.1 | Retro. Lenti |
| Sindbis virus | NC_001547.1 |  |
| Ebola Zaire | NC_002549.1 |  |
| Japanese Encephalitis virus | NC_001437.1 |  |

**Additional table 2. RNA and DNA datasets obtained from public databases**

| **Organism** | **Type of sample** | **Dataset ID** | **Source** |
| --- | --- | --- | --- |
| GRCh37( hg19) [release75] | RNA | GCA_000001405.14 | ftp://ftp.ensembl.org/pub/grch37/current/fasta/homo_sapiens/cdna/ |
| Monkey (M. mulatta_8.0.1) | RNA | GCA_000772875.3 | ftp://ftp.ensembl.org/pub/release-96/fasta/macaca_mulatta/cdna/ |
| B.taurus_UMD3.1 | RNA | GCA_000003055.3 | ftp://ftp.ensembl.org/pub/release-94/fasta/bos_taurus/cdna/ |

**Additional table 3. Primers and oligonucleotides used in this study**

| **Target** | **Primer or Oligo ID** | **Sequence (5**′ **🡪 3**′**)** | **Reference** |
| --- | --- | --- | --- |
| tax | RPX2 | CCGGCGCTGCTCTCATCCCGGT | Satou et al. PNAS 2016;113(11):3054-9 |
|  | RPX5 | GGCCGAACATAGTCCCCCAGAG |  |
| gag | gag_F | CAGAGGAAGATGCCCTCCTATT | Satou et al. PNAS 2016;113(11):3054-9 |
|  | gag_R | GTCAACCTGGGCTTTAATTACG |  |
| pol | pol_F | CAGCCCATTCGGCAAG | Satou et al. PNAS 2016;113(11):3054-9 |
|  | pol_R | TGAGAGTAGTAGTAGGTCCTCATGG |  |
| 18SrRNA | 18SrRNA_F | GTAACCCGTTGAACCCCATT | Satou et al. PNAS 2016;113(11):3054-9 |
|  | 18SrRNA_R | CCATCCAATCGGTAGTAGCG |  |
| ZC3HAV1 (ZAP) | huZAP_F | CCACATCTTCTAGGGTGGATGA |  |
|  | huZAP_R | CGTCCAGGTTTTACCAATAAGCA |  |
| TAP2 | TAP2_Ex4_F | CCAGGACCTCGGTTTCTTCC |  |
|  | TAP2_Ex5_R | ACCACTTTCACCAGGCTTCGC |  |

**Additional table 4. HIV-1 sequences we analyzed in this study**

| **HIV-1 RNA** | **ID** |
| --- | --- |
| **unspliced** | **HIV_unspliced** |
| **vif** | **HIV_vif** |
| **vpr** | **HIV_vpr_1.3i** |
| **vpu** | **HIV_env/vpu_1.5i** |
| **tat** | **HIV_tat_1.2.3.4.7** |
| **nef** | **HIV_nef_1.5.7** |
| **rev** | **HIV_rev_4c_1.4c.7** |

**IDs are described in the reference paper .**
